# Financing health system elements in Africa: A scoping review

**DOI:** 10.1371/journal.pone.0291371

**Published:** 2023-09-13

**Authors:** Humphrey Cyprian Karamagi, David Njuguna, Solyana Ngusbrhan Kidane, Herve Djossou, Hillary Kipchumba Kipruto, Aminata Binetou-Wahebine Seydi, Juliet Nabyonga-Orem, Diane Karenzi Muhongerwa, Kingsley Addai Frimpong, Benjamin Musembi Nganda

**Affiliations:** 1 Data Analytics and Knowledge Management, World Health Organization (WHO) Regional Office for Africa, Brazzaville, Republic of Congo; 2 Health Economist, Ministry of Health, Nairobi, Kenya; 3 Economic Planning Manager, Ministry of Health, Benin; 4 Universal Health Coverage–Life Course, WHO Regional Office for Africa, Brazzaville, Republic of the Congo; 5 Health Financing, Universal Health Coverage Life Course Cluster, World Health Organization, Regional Office for Africa, Brazzaville, Congo; Lorestan University of Medical Sciences, ISLAMIC REPUBLIC OF IRAN

## Abstract

Countries that are reforming their health systems to progress towards Universal Health Coverage (UHC) need to consider total resource requirements over the long term to plan for the implementation and sustainable financing of UHC. However, there is a lack of detailed conceptualization as to how the current health financing mechanisms interplay across health system elements. Thus, we aimed to generate evidence on how to utilize resources from different sources of funds in Africa. We conducted a scoping review of empirical research following the six-stage methodological framework for Scoping Review by Arksey & O’Malley and Levac, Colquhoun & O’Brien. We searched for published and grey literature in Medline, Cochrane Library, PubMed, WHO database, World bank and Google Scholar search engines databases and summarized data using a narrative approach, involving thematic syntheses and descriptive statistics. We included 156 studies out of 1,168 studies among which 13% were conceptual studies while 87% were empirical studies. These selected studies focused on the financing of the 13 health system elements. About 45% focused on service delivery, 13% on human resources, 5% on medical products, and 3% on infrastructure and governance. Studies reporting multiple health system elements were 8%, while health financing assessment frameworks was 23%. The publication years ranged from 1975 to 2021. While public sources were the most dominant form of financing, global documentation of health expenditure does not track funding on all the health system dimensions that informed the conceptual framework of this scoping review. There is a need to advocate for expenditure tracking for health systems, including intangibles. Further analysis would inform the development of a framework for assessing financing sources for health system elements based on efficiency, feasibility, sustainability, equity, and displacement.

## Introduction

Sustainable Development Goal 3 (SDG3) assures healthy lives and promote well-being for all, at all ages [1]. Universal health coverage (UHC) is a global health priority anchored within SDG3, as target 3.8. It is at the centre of current efforts to strengthen health systems and improve the level and distribution of health and health services [2]. It ensures financial risk protection, access to quality essential healthcare services, and access to safe, effective, quality, and affordable essential medicines and vaccines for all [3].

The SDGs and the Global Health Security Agenda (GHSA) are frameworks for guiding policy and program development to improve health globally [1]. The GHSA focuses specifically on health, and even more narrowly, on the threat of infectious diseases to global security [4]. Global public health security includes proactive and reactive activities that lower the danger and impact of acute public health events that endanger people’s health across geographical regions and international boundaries. Both the SDGs and the GHSA promote global public goods and require solidarity and cumulative action by all stakeholders including governments, donors, and civil society organizations [5]. Health security works on two levels, individual and population. Individually, health security considers the risks and threats, as well as underlying vulnerabilities, to both communicable and non-communicable diseases (NCDs). On the population level, health security intersects with state security [6, 7].

Accelerating progress on SDG 3 and the commitment to leave no one behind requires unified efforts to address the determinants of health, the health inequities or disparities such determinants perpetuate. Determinants of health may be biological, behavioural, socio-cultural, economic, ecological, environmental, or political [8]. There is an increased commitment to improving health and health equity through multi-sectoral approaches that address social, economic, and environmental, and political factors. In 2013, the review of social determinants and the health divide in the World Health Organization (WHO) European Region recommended developing more “partnerships at all levels of government that enable collaborative models of working, foster shared priorities between sectors and ensure accountability for equity [9].

The attainment of the SDGs requires substantial investments [10]. Moreover, the move towards Universal Health Coverage imply a growing need for innovative health financing models. Health financing encompasses mobilization, accumulation, efficient and effective allocation of funds to cover the health needs of the people, individually and collectively. It ensures funding is available and sets the right financial incentives to providers, to ensure all individuals are able to access effective, essential, quality health services. Moreover, it is critical for achieving UHC to raise adequate funds for health in ways that ensure people can use needed services and that they are protected from financial risk [11, 12]. By understanding the financing of health systems and services, programs and resources can complement the health financing mechanisms already in place, advocate for more funding of priority areas, and increase population access to available health services [13]. Africa makes up 16% of the world population and carries 23% of the global disease burden, yet it accounted for only 1% of total global health expenditures in 2015. In per capita terms, the rest of the world spends ten times more on health care than Africa [14].

Healthcare systems are funded through domestic funding from central government, local governments, private expenditure from individual households (out-of-pocket (OOP) expenditure), NGOs (including religious organizations and local philanthropies), private companies and external funding from donors [15]. Health care financing schemes are the main “building blocks” of the functional structure of a country’s health financing system, through which health services are paid for and obtained by people [16]. Health financing schemes raise revenues to pay for health care goods and service for the population they are covering. They receive transfers from the government, social insurance contributions, voluntary or compulsory prepayments (e.g. insurance premiums), other domestic and external revenues from abroad as part of development aid [17]. Social health insurance is financed out of social contributions payable by employees and employers. However, these schemes may also receive a varying proportion of their revenues from governmental transfers. The main sources of revenue for private health insurance are either compulsory or voluntary prepayments, which typically take the form of regular premium payments as part of an insurance contract [18].

Household health expenditures remains a major financial burden to households and health sectors in low- and middle-income countries (LMICs). More than 37% of Africa’s health spending comes from OOPs [19]. This burden has significant implications at the household level. For example, at least 11% of Africans experience catastrophic spending on health care every year, while as many as 38% delay or forgo health care due to high costs [14]. If the ambitious SDGs are to be reached in Africa, significant efforts must be made to change the current spending environment [14]. The role of the household out-of-pocket is of particular significance, as the financial burden is placed heavier on the poor. Such access barriers contribute to high burdens of preventable deaths. In addition, more than approximately 800 million people worldwide spend at least 10% of their income on healthcare through OOPs [20] which pushes millions of individuals further into poverty each year [21]. This can be attributed to the cost-sharing policy in public hospitals as seen in Kenya [22], low pre-payment mechanisms, and low expenditure on health by the government globally [23].

Strengthening of domestic financing is crucial to avoid OOPs and countries globally must increase their allocated spending on primary healthcare by at least 1% of their gross domestic product (GDP) for attainment of SDGs. In 2001, African Union countries pledged to spend at least 15 percent of their annual national budgets on health in the Abuja Declaration. Many African countries have limited capacity to raise public revenue due to the informal nature of their economies, rendering tax collection for social health insurance a challenge [24]. In the majority of WHO African Region Member States, external sources account for less than 20% of total health expenditure while out-of-pocket payments account for over 40% of the total health expenditure. In countries where user fees have been abolished or exemptions from fees extended to certain groups, there is a challenge in developing mechanisms for increasing funding for health from alternative sources [25].

Notably, these different sources of funding are usually focused on the different elements of the health system. Unfortunately, the forms of funding and funding levels for health systems’ building blocks are not well documented. It is unclear whether all the building blocks are funded adequately, achieve efficiency, equity and offer financial risk protection.

The WHO Regional Office for Africa (AFRO) calls for a shift in focus on investment in the six building blocks of the Framework of Actions [26], to a focus on having complex, dynamic systems that allow for the interplay across 13 elements. The current focus of investment in the 6 building blocks needs to evolve to a focus on having complex, dynamic systems that allow for an interplay across 13 elements, of which there are 3 hardware (staff, medical products, infrastructure), 4 software (delivery and governance processes, information, and financial management system) and 6 intangibles (values & norms, beliefs, practices, organizational culture, interests & networks, relationships & power) elements.

Aligned to the evolving needs of the population for attainment of UHC, health security and determinants of health, health systems need to re-pivot and focus on the strengthening system capacities spanning access, demand, quality of care and resilience. Furthermore, it is important to explore whether these common allocations of health funds present the most appropriate way to use the resources, and where better use can be attained. In this review, our main objective is to generate evidence on the most appropriate ways to utilize resources from different sources of funds, taking into account the health systems pillars, to maximize the triple health financing objectives of resource adequacy, efficiency, and equity.

## Methods

### Design

We conducted and reported a scoping review according to the (PRISMA) extension for Scoping Reviews (PRISMA-ScR) [27] checklist (S1 Appendix). This scoping review adopted the six-stage methodological framework for Scoping Review, adapted from Arksey & O’Malley (2005) [28] and Levac, Colquhoun & O’Brien (2010) [29]. This framework includes identifying the research question, searching for relevant studies, selecting studies, charting the data, collating, summarizing, and reporting the results, and consulting with stakeholders to inform or validate study

### Inclusion and exclusion criteria

We included studies and National health account (NHA) data sets reporting on health financing of 13 health system elements in Africa and also included studies up to the time of the literature search (December 31, 2021). We included: (1) Peer-reviewed articles that were published in the English and French languages, (2) Peer-reviewed articles reporting empirical research or conceptual papers on the financing of health system functions, (3) Peer-reviewed articles that were open access and [4] data sets for NHA of countries within the WHO African region.

### Search strategy

The databases searched included, Medline, Cochrane Library, PubMed, WHO database, World bank database and Google Scholar search engines. In addition to the database search as outlined above, we also undertook the following to identify key evidence for the review:

1. Liaison with topic experts.
2. Citation searching on papers included and other key papers identified by topic experts.
3. Scrutiny of reference lists of the included in primary studies and relevant systematic reviews.
4. Scrutiny of recent reviews of services and guideline documents for relevant peer-reviewed evidence.

The search strategy was structured as follows:

- Population = Studies reporting health systems financing globally
- Intervention = Health systems financing.
- Comparator = There was no comparator for this scoping review
- Outcomes = There were main outcomes included;
  - The current focus of different sources of health funds in relation to the 13 health system elements
  - Framework for assessing financing sources for health system elements
  - The effectiveness of the current ways the 13 health system elements are financed from the four sources of funding
  - Perceptions on how to improve targeting of the four sources of funds towards the 13 elements of the system for them to function effectively
  - Recommendations of more effective ways that current sources of funds can be focused towards the 13 health system elements in order to maximize resource adequacy, efficiency, and equity

The following search terms were used:

(“health financing” OR “financing” OR “funding”) AND (“health workforce” OR “human resources for health” OR Staff OR drugs OR medicine OR “medical products” OR “health infrastructure” OR “Service delivery” OR “governance” OR “information systems” OR “health investment” OR “Health system strengthening” OR “Universal Health Coverage” OR “health security” OR “common goods for health”). Additionally, we specifically retrieved and reviewed the latest available National Health Accounts (NHA) reports for 19 African countries.

### Selection process

We operationalized our inclusion criteria based on our Population, Intervention, Comparator and Outcome (PICO) elements. Five reviewers (HCK, ND, SNK, HD, HKK,) participated in the design of the knowledge synthesis. Two reviewers (HD and ND) participated in the development of the search strategy and the selection of eligible studies. HD searched for grey literature and ND searched for published literature. The study selection was done independently and checked by another (HKK).

### Data extraction

Two reviewers (HD, and ND) participated in the data extraction. HD extracted data from the grey literature, ND extracted data from published literature. Full-text articles that fit the inclusion criteria were extracted for country, NHA year, health system elements financed, elements, title, assessment criteria, criteria definitions and criteria indicators, author, publication year focus, and conceptual or theoretical nature of the study. The extracted data was entered into a Microsoft Excel matrix. All disagreements were re-examined jointly, and appropriate corrections made for all studies included in the review.

### Data analysis

All reviewers (HCK, EB, SNK, HB, ND, ABWS, JNO, KAF, DKM, BMN) contributed to the analysis or interpretation of the data. We summarized data using a narrative approach involving thematic syntheses and descriptive statistics. In addition, a thematic review of grey literature on health financing including the WHO global health expenditure database was also conducted. We conducted literature research and developed 5-criterion framework for assessing the alignment of health financing sources with the objectives of funding health system functions. We synthesized extracted data on health financing assessment frameworks, integrated recurrent and relevant elements of multiple frameworks, to develop a framework for assessing health financing sources (See Table 1). The team ensured that all the evidence synthesized had undergone methodological and expert scrutiny. To generate the trends in contributions of different health financing sources to total health expenditure in Africa, we extracted data from the GHED database on the contribution of public sources, private sources, out-of-pocket expenditure and external funding.

**Table 1:** Framework for assessing health financing sources.

Criteria	Definition	Link between financing source and the framework	Criteria Indicators
**Efficiency**	An efficient health financing source maximizes revenue collection, and enhances technical and allocative efficiency of the health system	Out-of-pocket payments are most inefficient ways of financing healthcare, because the pool is fragmented with no efficiency gains due to high running cost on the part of healthcare providers. Prepaid mandatory payments offer the greatest possibilities for equity, efficiency and sustainability, for pooling, cross-subsidies and equitable allocation. There are concerns regarding efficiency in the allocation of private funding between primary care and other health care needs. The private sector may not have a strong incentive to undertake provision of primary health care. In addition, the private sector’s full potential is yet to be developed due to weak institutional links between the government and the NGO sector. Geographical coverage by the private sector is limited due to inadequate incentives and burdensome regulations.	**Indicator 1:** Proportion of administrative costs for revenue collection
			**Indicator 2**: The revenue generation potential of the funding source
			**Indicator 3:** The extent to which the funding sources can be flexibility allocated across priorities
**Feasibility**	A feasible health financing source has political support and is aligned with the system’s capacity to implement the health financing mechanism	It is far more feasible to argue for an increased percentage share of total government expenditure for the health sector where overall government expenditure is increasing in real terms and even if the health sector’s percentage share does not increase, an increase in fiscal capacity will automatically translate into increased government spending on health. Out-of-pocket (OOP) payments are not feasible and have severe consequences for health care access and utilisation and are especially catastrophic for the poor	**Indicator 4**: The level of political acceptability and support
			**Indicator 5**: The level of administrative ease of revenue collection
**Sustainability**	A sustainable health financing source maintains predictable levels of funding over the medium to long term	Public domestic financing is a more predictable and sustainable source of financing for the provision of healthcare than donor funding. OOPs depend on the ability and willingness to pay for services by the users. This, coupled the impoverishing impact of OOP makes it highly unsustainable.	**Indicator 6**: whether revenues from the source are projected to remain at the same level or grow over the long term (Revenue growth rate by funding sources)
**Equity**	An equitable health financing source enhances equity in financial contributions and use of health services	Donor funds tend to be inequitable because funded activities are not implemented equitably and support is for specific services that may not benefit everyone. OOP worsens existing inequities in access to available health services because ability to pay determines access to healthcare. Hence, the richest have better access (in terms of range and quality) to healthcare than the poorest. Government spending on health, although not explicitly stated, is generally expected to benefit the poor more than the better-off	**Indicator 7**: The level of progressivity of financial contributions
			**Indicator 8**: The extent of risk cross-subsidization
			**Indicator 9**: The effect on financial risk protection
**Displacement**	A health financing source has a displacement effect when it causes a reduction or reallocation of other sources of funding to other priorities	Donor funding has been shown to have a displacement effect on public expenditure on health	**Indicator 10**: Whether the funding sources have a displacing effect on other sources of revenues

## Results

### Selection of studies

**Fig 1:**
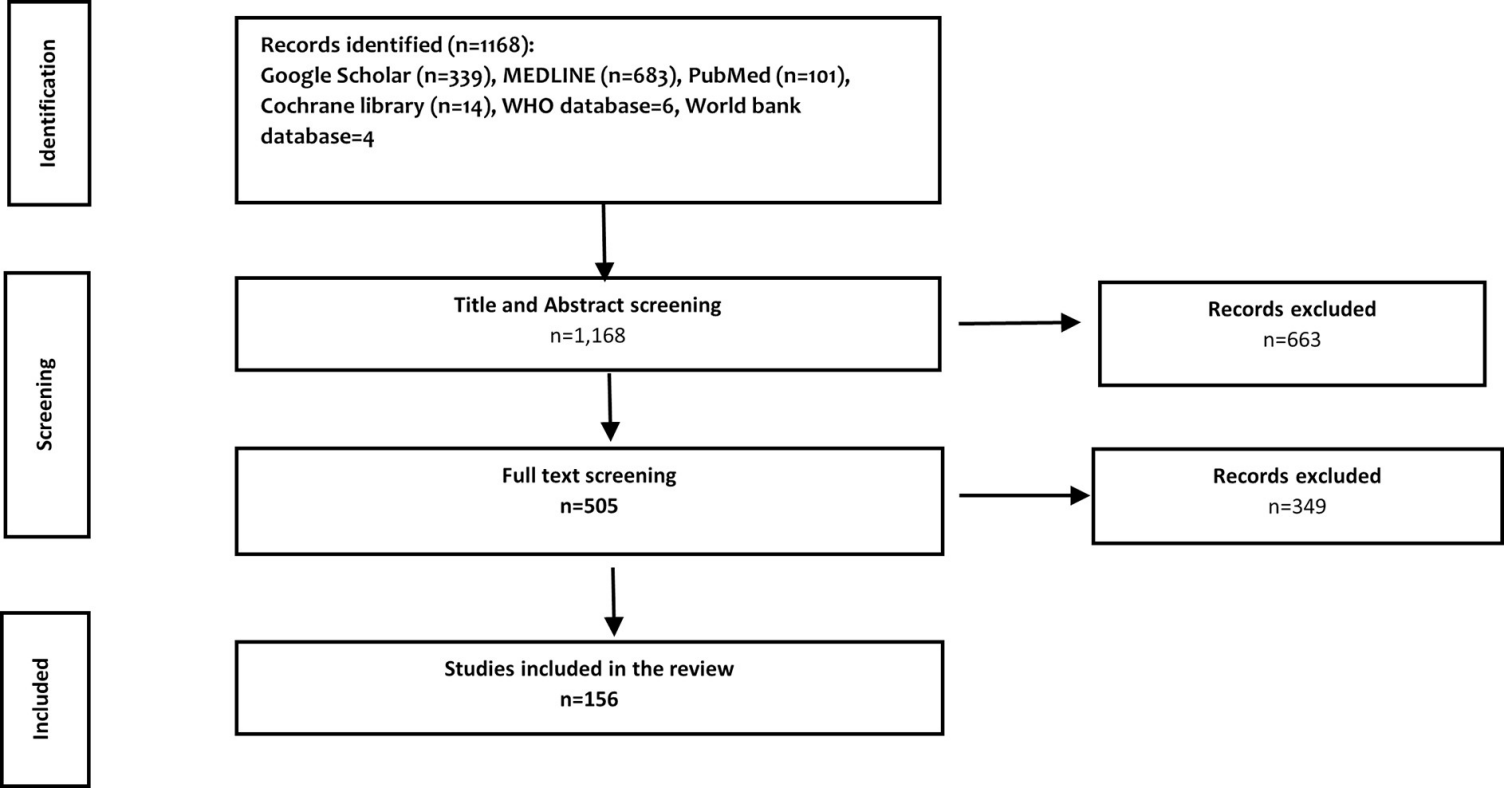
Literature selection flow diagram.

We identified a total of 1,168 papers. Of these, 663 articles were excluded after screening the titles and abstracts. An assessment of the full-text formats of the remaining 505 papers resulted in a further 349 exclusions. A total of 156 studies were finally included in the review (Fig 1). Additionally, we specifically retrieved and reviewed the latest available National Health Accounts (NHA) reports for 19 African countries between 2000 to 2019.

### Characteristics of included studies

Among the retrieved studies, 13% were conceptual studies while 87% were empirical studies. Empirical studies report verifiable evidence by observation or experience rather than theory or pure logic. Conceptual studies report abstract ideas or concepts and do not particularly involve any practical experimentation [30]. With regards to the focus of the papers, 45% of selected papers focused on service delivery, 13% on human resources for health, 5% on medical products, and 3% focused on infrastructure and governance. Papers that focused on multiple health system elements contributed 8% to the total selected papers. With regards to the NHA datasets, the health expenditure tracking focused on tangible hardware elements and some tangible software elements. Funding of intangible elements is not tracked. Most countries do not have accurate estimates of the factors of provision that would have been ideal in understanding the landscape for financing the elements in totality. Also, NHA data currently does not track health expenditures on health security. Recently there has been efforts to estimate health security as well as primary health care as a result of an in-depth analysis. Health financing assessment frameworks were presented in 23% of the selected papers. The publication years ranged from 1975 to 2021. A total of six studies were published between the years 1975–1979. Likewise, six studies were published between the years 1983 and 1987. Thirty-two (32) studies were published between 1991 and 1979, while 31 studies were published between the years 2000 and 2010. The majority of studies included (91) were published between 2011 and 2020 while 5 studies were published in 2021. The retrieved studies had a mix of qualitative, quantitative, and mixed methods, while some were conceptual studies.

### The current focus of different sources of health funds in relation to the 13 health system elements

**Fig 2:**
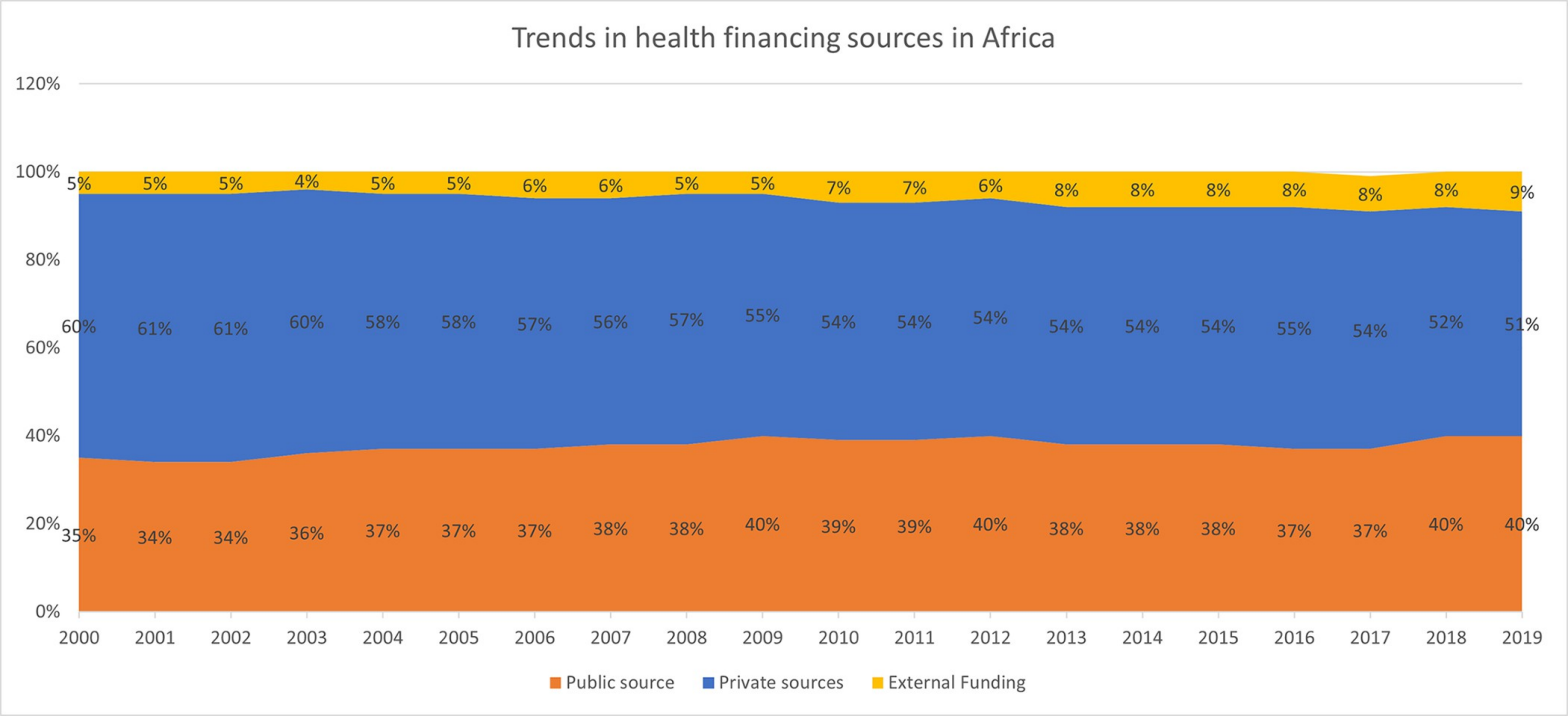
Trends in contributions of health financing sources to total health expenditure in Africa (Source: WHO GHED database 2000–2019) [19].

Fig 2 outlines an analysis of the contribution of different funding sources to Current Health Expenditure (CHE) in Africa over 20 years, using data from the WHO Global Health Expenditure Database (GHED) that provides comparable data on health expenditure for 192 countries over the past 20 years. The database is open access and contributes to a better understanding of countries’ spending on health, government, insurance companies, households and donors’ contribution to health spending, primary health care (PHC) expenditure, expenditure on specific health services and different diseases and conditions.

Several issues emerge from these analyses. First, private sources of funding contribute the highest proportion of total health expenditure, although this has decreased from 60% in 2000 to 51% in 2019. It remains a substantial contributor to total health expenditure in Africa despite the commitment by countries to move towards UHC by, among others, scaling up prepayment health financing mechanisms. Second, the public source has increased from 35% (2000) to 40% (2009), and decreased until 2017, with slight rise observed in 2019 (40%). Third, external funding has increased over the last decade, from 5% to 9% between 2000 and 2019.

**Fig 3:**
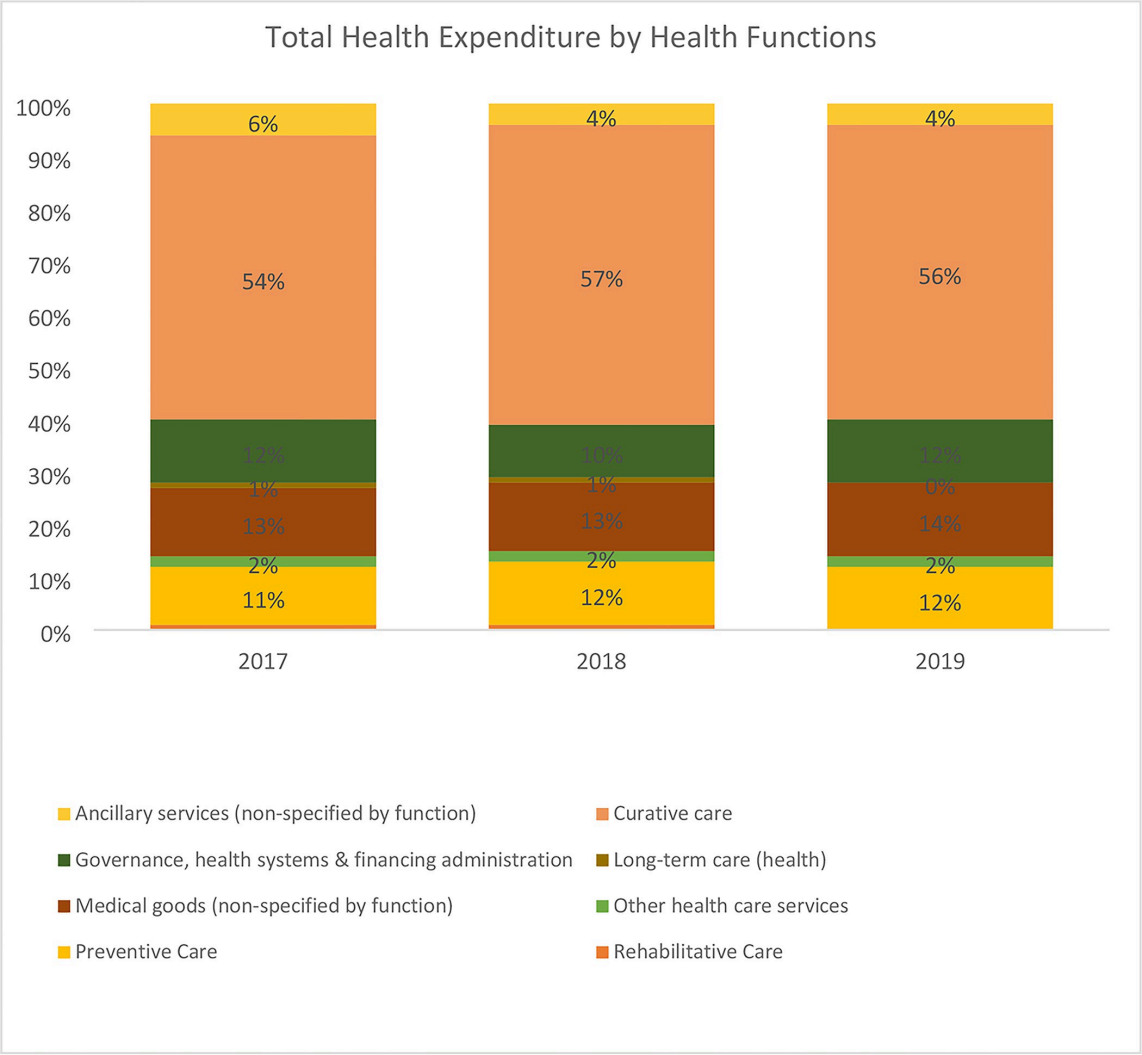
Proportion of total health expenditure by health system function (Source: WHO GHED database 2017–2019) [19].

An analysis of total health expenditure by health system functions reveals that the GHED tracks funding for only three out of the 13 health system elements. These include governance, medical goods, and service delivery (preventive, curative, long-term, rehabilitative, ancillary, and other healthcare services). The findings reveal that service delivery received the highest share of health sector funding in Africa between this period, followed by governance and administration and health commodities. An assessment of expenditures on health system functions by funding sources in Africa reveals a similar pattern. Most of the funding is allocated to service delivery followed by governance and administration, and lastly health commodities (Fig 3). The exception is on private and out-of-pocket expenditure where health commodities come second to service delivery. This analysis does not separate private prepaid expenditure from out-of-pocket expenditure since the WHO health expenditure database does not provide that to this level of disaggregation.

**Fig 4:**
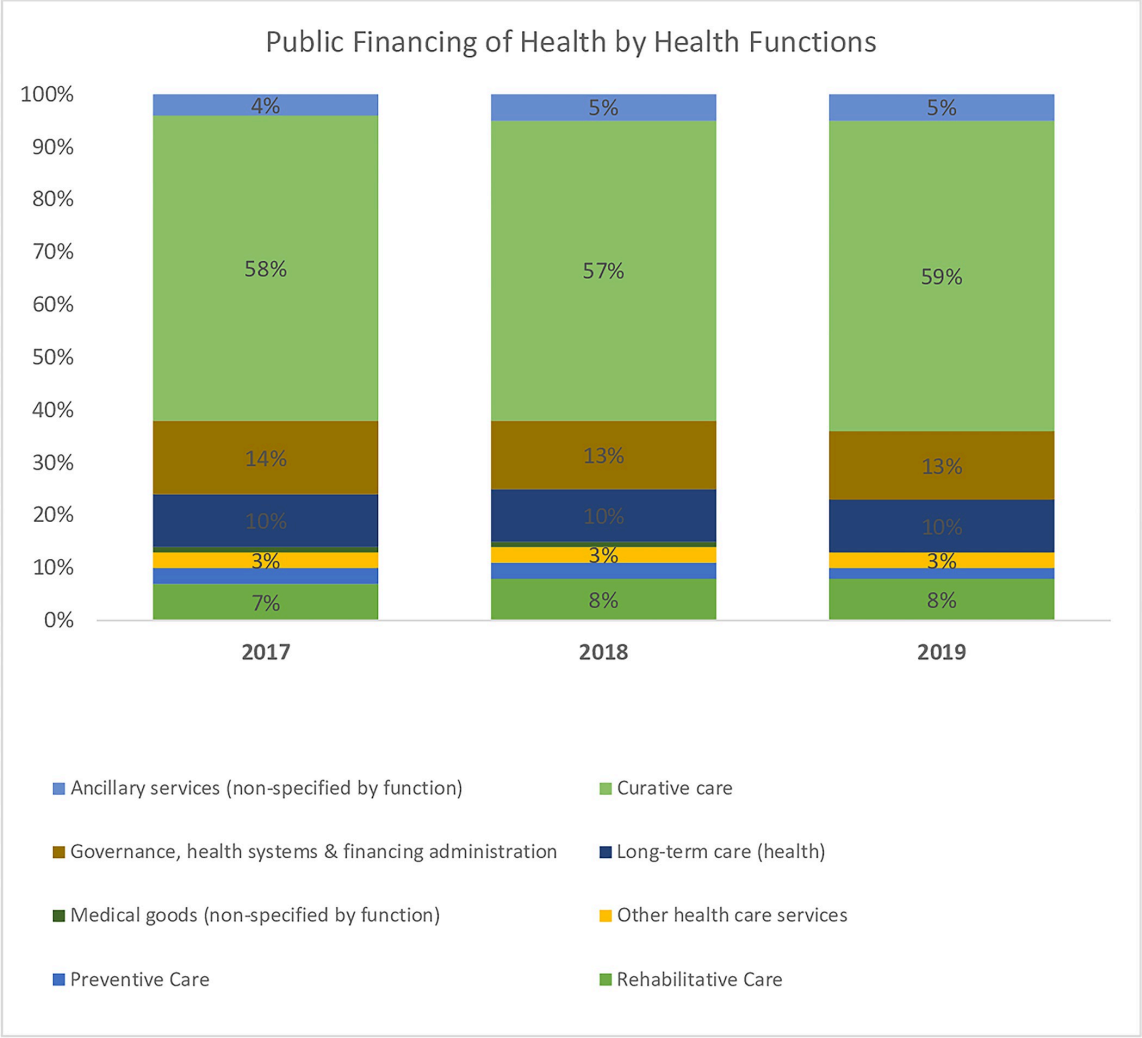
Funds and elements map.

**Fig 5:**
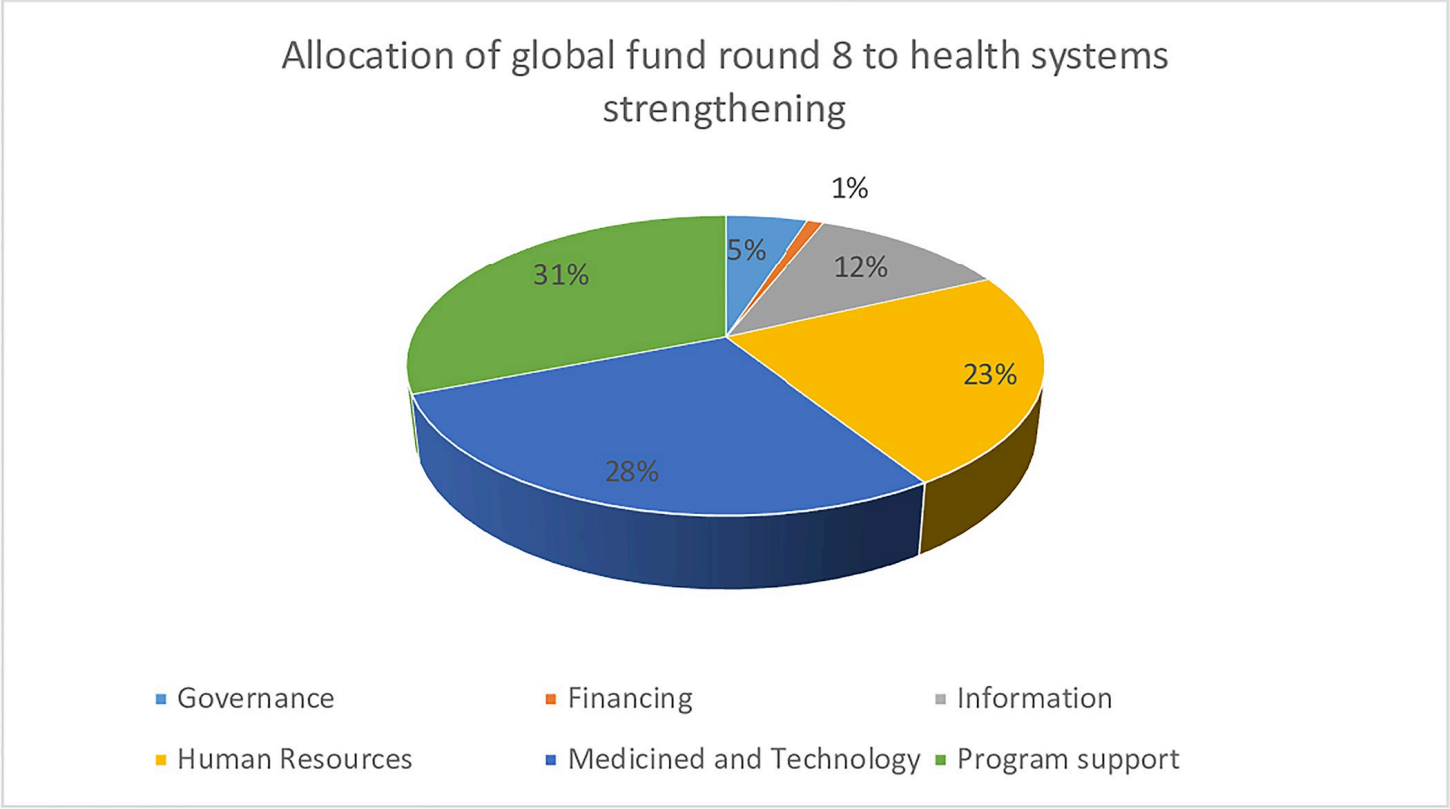
Resource allocation of global fund round 8 (Warren et al. 2013) [31].

Funding from all four sources was allocated across the health system functions. This is in line with the expectations of a system that pools and allocates funds across health sector priorities. It is worth noting that private (excluding OOP) and OOP tend to focus on medical products and service delivery (See Fig 4). Moreover, while external funding was initially focused on vertical service delivery, there is an increased focus on health system strengthening. However, a deeper assessment of health system investments by external funders shows that the bulk of their health system spending is channelled within vertical disease programs, with little going to system-wide investments. For instance, 37% of The Global Fund to Fight AIDS, Tuberculosis and Malaria was spent on health system strengthening, and approximately 60% of this was invested in disease-specific system support (Fig 5).

**Fig 6:**
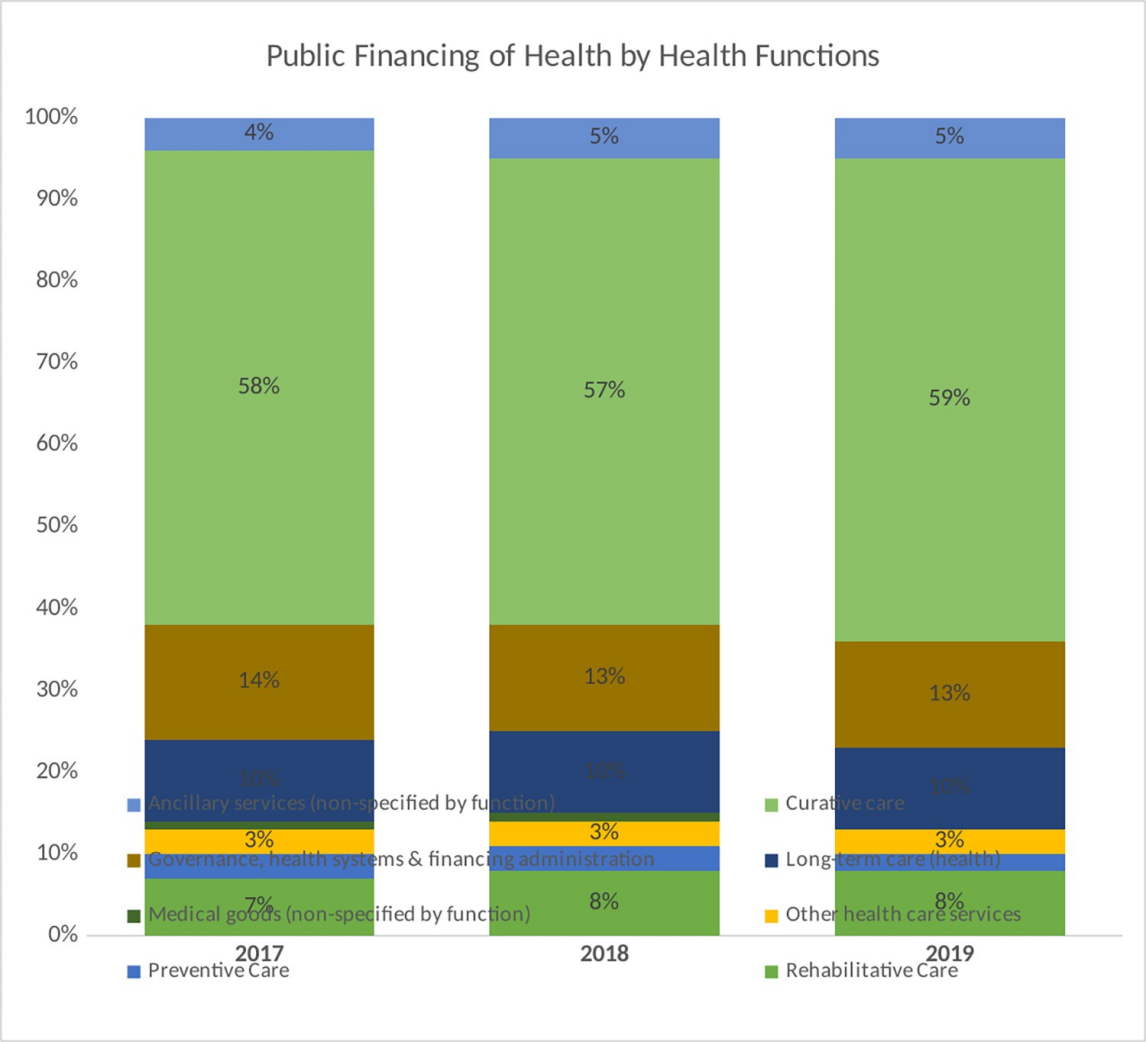
Expenditure on health system functions from public sources of funding in Africa (Source: WHO GHED database 2017–2019) [19].

**Fig 7:**
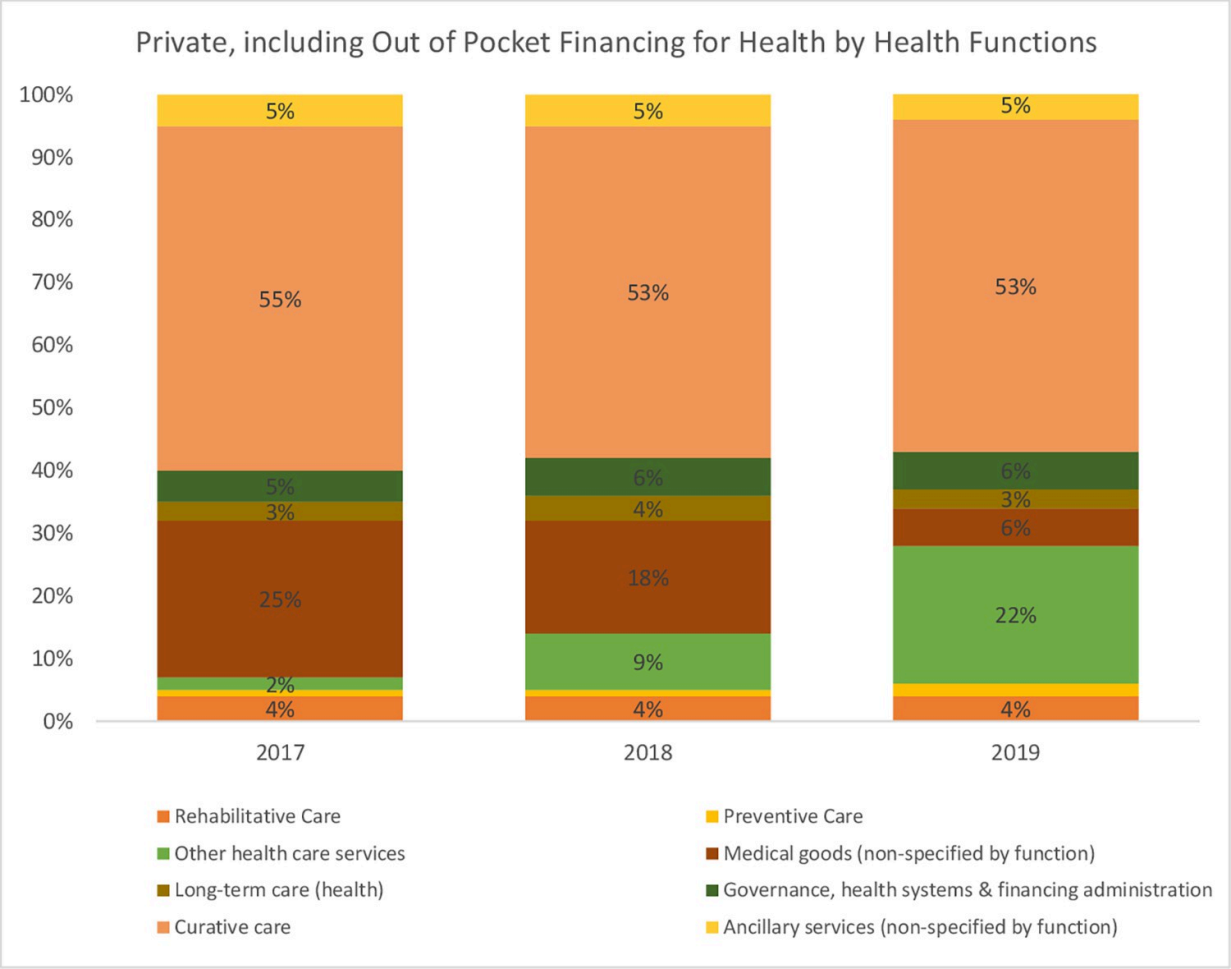
Expenditure on health system functions from private sources of funding in Africa (Source: WHO GHED database 2017–2019) [19].

**Fig 8:**
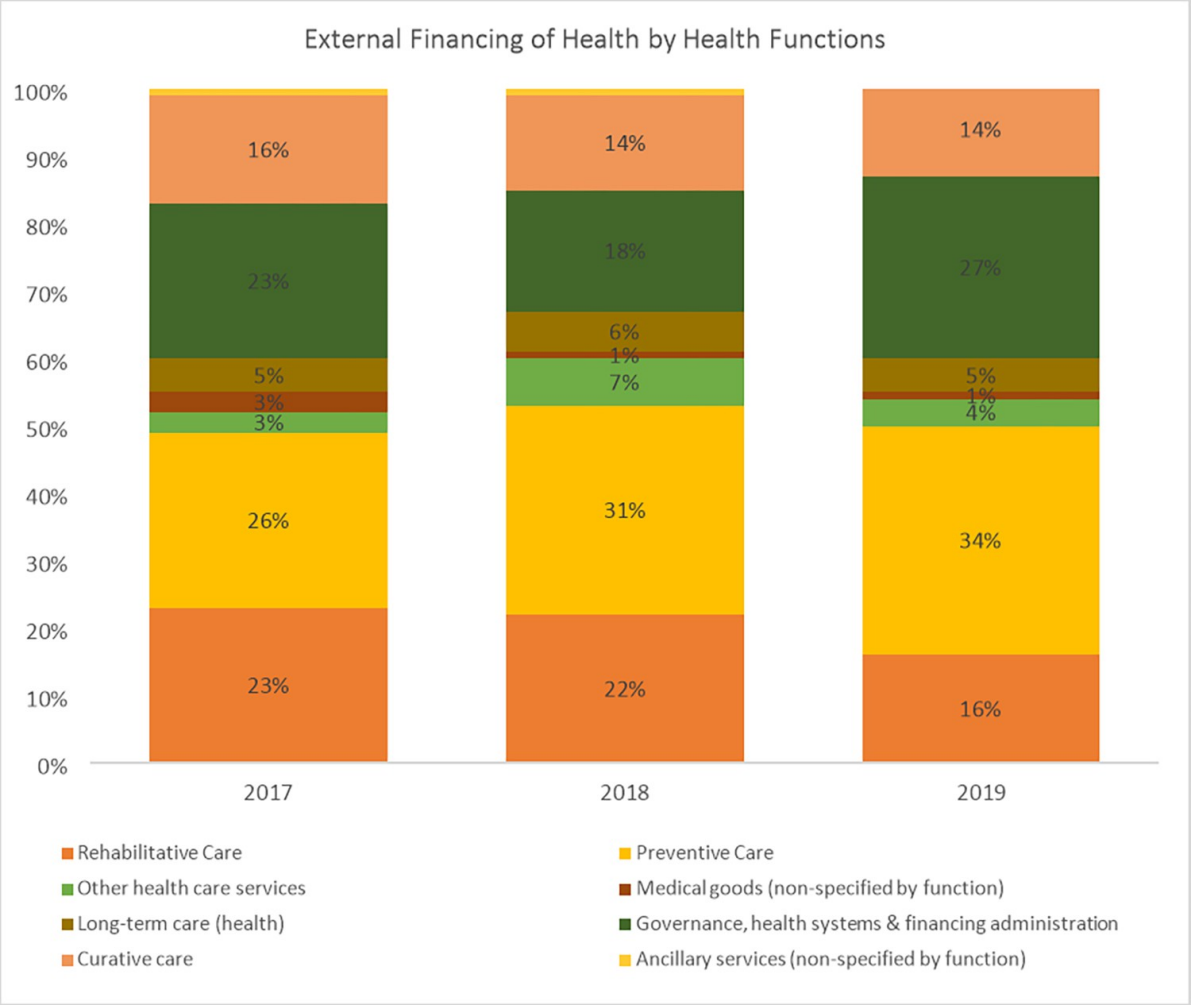
Expenditure on health system functions from external/donor sources of funding in Africa (Source: WHO GHED database 2017–2019) [19].

An assessment of external funding investments reveals a bias in allocation across health system elements. The Global Fund funding (round 8) shows that around 82% of health systems strengthening funding was allocated to service delivery, human resources, and medicines and technology. However, governance, financing, and information building blocks received relatively low funding (See Figs 6–8).

Global documentation of health expenditure does not track funding on all 13 dimensions that informed the conceptual framework of this assignment. There is evidence of tracking spending on health system tangible hardware (health commodities) and tangible software (governance, financial administration, and service delivery). Global tracking of funding emphasizes service delivery and disaggregates it into curative, preventive, and rehabilitative or palliative care while focusing on primary healthcare (PHC). However, it does not track expenditure on health security, making it difficult to assess the prioritization of health security by health systems. Expenditures on intangible aspects of the health system are not tracked, which is not surprising given the difficulty in tracking and quantifying such expenditures.

### Framework for assessing financing sources for health system elements

While some authors were explicit about proposing assessment frameworks or criteria, some were implicit in the sense that they applied criteria without formally proposing them as part of a framework for health financing assessments. Further, frameworks and criteria typically focused on the entire health financing function rather than the revenue mobilization function, which is the focus of this assignment. Focusing on recurrent themes and suitability of criteria to assess funding sources, we propose a funding source assessment framework that is comprised of five criteria (efficiency, feasibility, sustainability, equity and displacement), and 10 indicators (See annex 3 and Table 1)

### The effectiveness of sources of funding for the 13 health system elements

**Fig 9:**
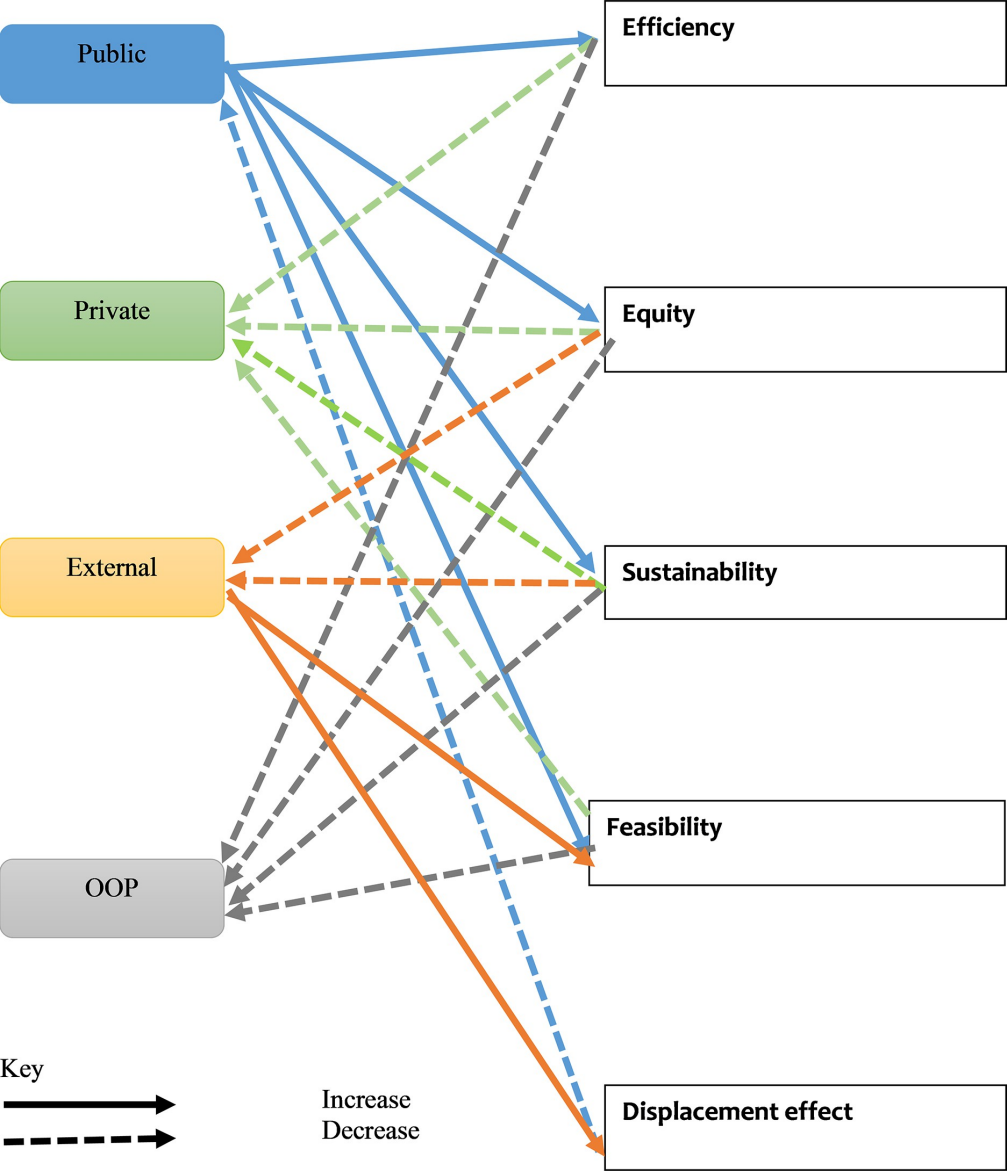
Application of framework by funding source.

The framework developed in Table 1 has been used to assess the suitability of funding sources and arrangements for health system elements. A summary of this assessment in provided in Table 2 and Fig 9.

**Table 2:** Performance of health financing sources against assessment criteria.

Source of funding/assessment criteria	Efficiency	Equity	Sustainability	Feasibility	Displacement effect
Public sources	+	+	+	+	-
Private sources	-	-	-	-	ND
Out of pocket funding	-	-	-	-	ND
External funding	ND	-	-	+	+

+ enhances;–reduces; ND: no data on criteria

### Emerging perceptions on improving sources of funds towards the 13 health systems elements

#### Human resources for health

Donors need to work with governments to understand how national and donor policies could impact program sustainability, integration, and coordination to maximize human resources for health (HRH) investments and improve health outcomes. Innovative financing mechanisms should work for the health system and the overall health workforce. Better coordinated financing of HRH training and activities will lead to less duplication, lower costs for training programs and strengthened national training programs that focus on long-term, pre-service training rather than short-term, ad hoc trainings. There is a need to establish a regulatory framework that supports resource mobilization for increased HRH capacity whilst considering proper assessment and forecasting. The framework should also determine the cadres based on needs, the number of beneficiaries eligible for funding each year, and a revolving fund to ensure sustainability. Increased investment by donors and governments in the basic pre-service training is required. There is also a need to increase dialogue, intensify health education, and cooperate to enhance health personnel capacity. Governments need to regulate staff transfers and increase health budget allocation.

#### Service delivery

To define clear roles and responsibilities of relevant parties, outline the nature and timeline of deliverables, and draw a clear scale-up and sustainability plan, it is crucial to map areas and sectors, where the government needs support from partners and non-governmental organizations (NGOs). For allocative efficiency, guided by the national priorities, harmonization of external and domestic spending as well as expenditure allocation, are crucial. Global-health initiatives should have a proactive and balanced investment approach to concurrently strengthen PHC systems, achieve programme targets, and sustain the gains, in resource-poor settings. Besides, increased coverage of prepaid health financing mechanisms would reduce over-reliance on potentially catastrophic and impoverishing out-of-pocket payments. Policy interventions aimed at financial risk pooling mechanisms are crucial to reducing the intensity and impact of OOP payments among vulnerable households. Governments should track how resources are allocated sub-nationally to maximize equity and ensure allocations are commensurate to health needs. This makes countries and donors accountable for their commitments to service delivery. More attention to the efficiency in using available resources is required, as savings can go a long way in decreasing the funding gaps and increasing impact.

#### Infrastructure

Countries need to develop health policies that address inequities and health financing models, that optimize utilization of health resources and strengthen health infrastructure. Further research should be carried out to determine the sustainability of the gains made. Additionally, an evaluation of donor-funded health system infrastructure in comparison to non-funded health system infrastructure is required.

#### Information management systems

There is a need to invest in coordinated efforts towards strengthening monitoring and evaluation systems. The donor-supported programs need to integrate parallel reporting systems with the national/district health information system used in countries, to ensure flexible, reliable, and robust systems.

#### Governance

It is vital to explore how the financing of civil society by donors influence policy agenda-setting, institutional innovations for increased civic participation in health governance and accountability to citizens.

#### Medical products

Tracking changes in health financing patterns across time and benchmarking against global trends is vital to addressing missed opportunities, ensuring access to medicines and high-quality services, and pursuing UHC. Improving engagement will help governments with limited resources to better take advantage of the private sector capacity to meet access and equity objectives.

## Discussion

This study highlights that private source of funding contributes to the highest proportion of total health expenditure. However, private sources contribution has decreased by 9% between the years 2000 and 2019. There has also been an increased allocation in domestic/public sources by 5% between 2000 and 2019. The increased allocation of domestic resources for health is consistent with the findings of [32] which reported that domestic resources exceeded funds provided by donors and accounted for the majority of global HIV funding (57%), totalling US$ 10.6 billion [32]. The amount of domestic investment LMICs are making in responding to HIV has grown by 50% between 2010 and 2019 [33]. Public financing provides the greatest improvement in health system functionality (access, quality, resilience and demand) compared with external and out-of-pocket financing [34]. Furthermore, public expenditure on health is a determinant for the achievement of UHC [35]. The extent to which public spending on health aligns with health system goals is dependent on the financing arrangements. Public funding is mobilized either through taxation or social health insurance contributions. Taxation is more efficient and sustainable compared to social health insurance contributions [36, 37]. While generation taxation is typically progressive and equitable in Africa, indirect taxes such as value-added tax (VAT) have mixed effects. For instance, while VAT is progressive in Ghana [36], it was regressive in Kenya [38]. Increasing domestic tax revenues is also integral to achieving UHC, particularly in countries with low tax bases. Pro-poor taxes on profits and capital gains are essential to expanding health coverage without the adverse associations with health outcomes observed for higher consumption taxes. Progressive tax policies within a pro-poor framework are also required to accelerate progress towards achieving major international health goals. Governments should increase earmarked revenues for health and broaden the VAT base. Continued efforts to exempt medicines from VAT, to reduce the cost of care for the population, are also critical. Although challenging for LMICs, the shift towards domestic funding has fostered ownership and accountability in the implementation of the national HIV response, which has increased their sustainability [39]. Simultaneously, it is critical to address other system issues and restore public confidence in government-supported programs.

Despite the commitment by countries to move towards UHC, the findings of this study also indicate that out-of-pocket payments remain a substantial contributor to total health expenditure in Africa. Unfortunately, out-of-pocket expenditure has a negatively correlation with health system functionality [34]. They are fragmented and hence inefficient [40] regressive and do not cross-subsidize risk [37]. They are also responsible for catastrophic health expenditures and impoverishment, making them an inequitable source of funding.

This calls for increased coverage of prepaid health financing mechanisms, which reduces over-reliance on potentially catastrophic and impoverishing OOP payments. The scale-up of prepayment mechanisms is an important part of the overall strategy of UHC and an essential step in promoting access to healthcare, while health insurance reforms continue to be deliberated [41].

There is a need to emphasize the crucial authority vested in the government, to support the move towards compulsory (rather than voluntary) prepayments for healthcare and pooling of funds to purchase healthcare. Moving toward compulsory payments enhances redistributive capacity and allows cross-subsidization across the population. Safety nets for the poor are needed to reduce the burden of spending by households. Policy interventions aimed at financial risk pooling mechanisms are crucial to reducing the intensity and impact of OOP payments among vulnerable households [42]. Moreover, countries need to ensure adequate allocation of resources through strengthening legal and institutional frameworks for resource allocation, formalizing the budgetary processes and resource allocation procedures [41].

External funding has had a significant impact on expanding access to critical health services in LMICs [43]. For instance, the Global Alliance for Vaccines and Immunization (GAVI) on expanding access to immunization [44], the U.S. President’s Emergency Plan for AIDS Relief (PEPFAR), and the Global Fund on HIV/AIDS, TB, and malaria [45]. External funding has also affected the efficiency of health systems [46]. Vertical programs have compromised the coordination of overall health systems as integration with the rest of the system has been limited [46–49]. This has resulted in duplication of functions such as procurement, monitoring and evaluation, information systems, and drained health workers from other services, due to the added financial incentives for health workers in these-donor funded programs [45]. The fragmented and vertical funding arrangements are exacerbated challenges already experienced by governments in tracking their resources [46].

Public health priorities have been affected since donor preferences are sometimes at odds with local priorities [46]. An assessment of Global Fund rounds one to seven funding found that investments in human resources or health were not coordinated with the rest of the system [50]. Furthermore, external funding has been fragmented, with little coordination across different donors. While there have been efforts to coordinate donor funding at the country level through the sector wide approaches (SWAps) [51], and at the global level through initiatives such as the Health Systems Funding Platform, the extent to which these initiatives have been successful is debatable [52–55]. The narrow focus, combined with the poor integration and coordination of external funding has therefore compromised the efficiency of health systems [50, 55].

Donor funding cannot be considered sustainable in the long term since there are plans by major donors such as GAVI and PEPFER to progressively exit as countries graduate to middle-income status [56]. It is evident that donor funding has increased between 2000 and 2013 but has stagnated since then. Likewise, the Kenya National Health Account that showed that the donor contribution reduced to 22% of current health expenditure in fiscal year 2015/16, down from 25.5% in fiscal year 2012/13 [57]. As economies grow from low- to middle-income countries, there is an anticipated decline in development assistance for health. Consequently, LMICs benefiting from donor investments must prepare for the eventual scale down and transition of external funds and technical support. Transitioning these programs while sustaining effective coverage of the essential services will require governments to effectively plan, implement, and monitor activities effectively as part of their routine budget and public financial management (PFM) processes [58]. A locally formulated donor transition readiness plan is required to pinpoint particular dimensions of a country’s health financing system that are most at-risk in an aid transition, not only from funding perspective but also from a programmatic point of view. The transition plan will also mitigate disruptions to care or increases in financial hardship to service users that are anticipated in case of sudden shocks to the financing of these areas [59]. This will require donors, countries, and technical partners to agree and address several issues together. Successful transition will require health plans to be integrated and aligned with overall economic and fiscal policies.

Private funding provides limited financial protection, generates healthcare inequities and has poor portability of entitlements. Consequently, it achieves limited population coverage because of the challenge of scaling up voluntary contributions in LMICs, that are characterized by high poverty and informality, and hence inefficient and unsustainable, rendering it inequitable [60]. Public-private partnerships (PPPs) leverage private sector resources to further public health goals [61]. PPPs in the health sector have focused on most health system functions. For instance, PPPs have been used to develop human resource capacity through training programs [62, 63], finance hospital care [64] and health care commodities [65]. There are mixed findings on the impact of PPPs in the health sector. While they have been shown to be successful in scaling up the provision of services such as laboratory services [66] and delivery of essential commodities [65], some PPPs have been unsustainable, inefficient [67, 68], and inequitable [69]. A key challenge with PPPs in Africa is the weak regulatory and policy environment, such that often PPP arrangements are often informal, and varying capacity of government to negotiate and structure PPPs terms are aligned with health system goals or public goods. This leads to inappropriate distribution of risk and reward leading to moral hazards [70, 71].

The private sector is well-positioned to contribute to the UHC and already provides health products and services to the population. It can raise finances required for UHC by contributing to innovative finance models and tools, engaging constructively in dialogue on corporate taxation, and supporting governments to articulate the business case for investing in health and UHC. It can also develop, test and scale-up innovative business models that align with UHC goals, especially approaches that drive greater and more equitable access, quality and sustainability of health product and services. However, there is also a need to look beyond immediate results and take a long-term approach that promotes the development of resilient and sustainable health systems [72]. Finally, donor funding has been shown to be fungible. Studies on foreign aid have largely established empirically that aid is usually fungible, which often raises questions about the effectiveness of aid given to those developing countries where fungibility of development assistance exists [73].

The development of a universal framework for health financing research is also essential to support the push towards global sustainable health financing. There is a need to prioritize health in domestic budgets and to better exploit economic growth to increase health spending as countries transition from external aid. Improving the performance of tax systems will be a step in the right direction, with possible long-term gains to the health sector [74]. Furthermore, advocates for health investments should use healthcare as political capital by identifying potential allies or highlighting how investing in health could help further a government’s broader agenda. Governments can also set priorities that better respond to the health needs of their citizens and help them make a case for further increasing their health budgets relative to other national priorities. Forthcoming health financing reform agendas must also incorporate a strategy for getting data used in the design of financial risk protection [75].

There is also a need to cut ineffective spending and waste to produce significant savings. For policy makers struggling to cope with ever-growing health care expenditure, all opportunities to move towards a more value-based health care system must be pursued. Wasteful spending on health relates to services and processes that are either harmful or do not deliver benefits. It also includes excess costs that could be avoided by replacement with cheaper alternatives with identical or better benefits [76]. A particular area for improvement cited is the alignment of grant development, budgeting, and reporting with health sector planning, budgeting, and reporting cycles [77]. Historically, line-item budgets were the predominant method of presenting government budgets. Line-item budgeting was criticized for holding public agencies accountable for only what was spent and not what had been achieved from the expenditure [78]. In contrast, Program-Based Budgets are organized around programs and sub-programs, with funds allocation linked to technical priorities and outcomes. It can link sector level technical priorities with budgeting; and enhance transparency, openness, and efficient use of public resources through public participation [79].

### Effective ways current sources of funds can be focused towards the 13 health system elements in order to maximize resource adequacy, efficiency, and equity

#### Improved coordination of sources of funding

A better coordinated and streamlined financing architecture, which is linked to clear results and backed by accountability and reporting mechanisms, will lead to minimised duplication of efforts, and reduce unreasonably high transaction costs for governments and other financing partners. Moreover, greater alignment of financial roadmaps, based on costed health systems inputs/elements, can improve leverage prospects for healthcare investors, which in return can enhance efficiency and effectiveness of the health investments made.

#### Prioritize funding for health security and other common goods for health (CGH)

UHC requires governments to invest in health security and common goods for health, since they represent services that are the backbone of all health systems and, more broadly, societies. CGH fall under five categories: policy and coordination; taxes and subsidies; regulation and legislation; information, analysis and communication; and population services. To ensure global health security, international resources must be pooled predictably and sustainably to provide stable and sufficient additional financing, and increase domestic financing on pandemic preparedness and response. The CGH financing agenda should ideally be integrated into health financing and national budgets dialogues, accompanied by regulation, monitoring, and accountability measures, to ensure political and funding commitments result in the provision of CGH. The decision to advance government action on common goods requires an articulation of the defined problem, a well-defined strategy for addressing the problem and leadership that is backed by key stakeholders.

#### Prioritize funding for health system tangible software

Service delivery, governance processes, information systems, and financial management systems are central to improving health sector performance and achieving UHC. Investment cases can be developed to enhance strategic investments by government bodies, for increased funding and improved efficiency in spending. These can be further augmented by articulating succinctly the potential impact on health outcomes and economic growth. Consequently, this would allow stakeholders and advocates to rally behind a single message and funding for the health system tangible software elements.

#### Incentivize health system intangibles

Incentivizing health system intangibles requires performance management systems or processes that help define and track progress against the health system goals and national development plans. It is anchored on empowering the health workforce with the opportunities and resources that create strong engagement, as well as the formal and informal recognition efforts that help satisfy their needs for achievement.

#### Invest in nurturing health system capacity for intangible elements

Health systems are value-driven and consequently, strive for excellence in meeting the needs of their patients in a caring, quality-focused, and safe environment. It is important to understand the core values of the system as they interlink with all other health system elements and are responsible for their propagation. Building conducive cultures and collective corporate values for the health system is a key ingredient in nurturing health system capacity.

Evidence from this review also shows that both tangible and intangible software elements play as important of a role in health system performance as health system hardware. LMIC governments should prioritize funding for health system software as part of health system reforms. There is a need to incentivize and nurture health system intangibles given the difficulty in directly funding them. This can be achieved by explicitly recognizing their value, including them as part of performance management for staff and health system organizations, and supporting funding efforts to develop capacities, e.g., soft skills, coaching and mentorship of health system leaders and managers.

### Study limitations

GHED uses the available NHAs from countries and then projects based on the funding landscape. This does not factor financing arrangement changes thereby shifting funding to different elements. The NHA report here does not track spending on intangible software elements and health security. The NHA (SHA) provides a detailed approach of tracking factors of provision (inputs for production of healthcare) but countries continue reporting more on the other dimensions other than factors of provision which ideally would be useful when tracking for the 13 health system elements.

## Conclusions

The current focus of different funding sources for the 13 health system elements is skewed, with public sources being the most significant contributor and the private sector lagging, pointing to opportunities for more balanced investments. Moreover, there is not any preference to fund specific health system functions. Funding from all four sources is allocated across all health system functions. Besides, global documentation of health expenditure does not track funding on all 13 dimensions that informed the conceptual framework of this assignment. While there is expenditure for health system tangible hardware and tangible software elements, no study reports tracking spending on health system intangibles. Investments should be made on tracking health systems intangibles since health system strengthening depends on inter-agency collaboration and country commitment along with concerted partnership among all the stakeholders working in the health sector and whose work affects the health system, whether directly or indirectly. Overall, these findings point towards a target of government spending on health of at least 5% of GDP for progressing towards UHC.

## Supporting information

S1 Checklist Preferred Reporting Items for Systematic reviews and Meta-Analyses extension for Scoping Reviews (PRISMA-ScR) checklist. (PDF)

S1 Appendix List of reviewed literature. (XLSX)

S1 File Health financing frameworks and framework elements identified in literature. (DOCX)
