# Supplementary material for: Financing health system elements in Africa: A scoping review
Source: PLoS One. 2023 Sep 13;18(9):e0291371. doi: 10.1371/journal.pone.0291371 (PMC10499258; doi:10.1371/journal.pone.0291371)
Supplement: S1 File — (DOCX) [file pone.0291371.s003.docx]

S3. Health financing frameworks and framework elements identified in literature

| Reference in chronological order | Assessment criteria | | | | | |
| --- | --- | --- | --- | --- | --- | --- |
|  | **Efficiency** | **Equity** | **Sustainability** | **Feasibility** | **Fungibility** | **Others** |
| World Bank (1975) | √ |  |  |  |  |  |
| WHO (1978) | √ |  |  | √ |  |  |
| Zschock (1979) | √ | √ |  |  | √ | - Impact on healthcare |
| Griffiths and Mills 1983 | √ |  | √ | √ | √ | - The impact on health service utilization and provision - Effects on the economy |
| Hoare and Mills 1986 | √ | √ |  |  | √ | - Effects on supply and provision of services |
| World Bank (1987) | √ | √ |  |  |  |  |
| Van Balen and Mercenier (1991) |  |  |  | √ |  | - Social solidarity - Contribution to continuity of care |
| Parker and Knippenberg (1991) | √ |  |  |  |  | - responsible and empowered local communities |
| Hsiao (1992) | √ | √ |  | √ |  |  |
| Carrin & Vereecke 1992 | √ | √ |  |  |  |  |
| Green 1992 |  | √ |  | √ |  | - Effect on service provision - Participation in decision making - Effect on multisectoral action |
| Barnum & Kutzin (1993) | √ | √ |  | √ |  |  |
| World Bank 1993 | √ | √ | √ | √ |  | - Impact on healthcare utilization |
| WHO (1993) | √ | √ | √ | √ |  | - Impact on health status |
| Berman & Chawla (1995) | √ |  |  | √ |  | - Accountability - Quality of care |
| Shaw & Griffin (1995) | √ | √ |  | √ |  | - Public-private sector collaboration |
| Nolan & Turbat (1995) | √ | √ |  | √ |  |  |
| Creese & Kutzin (1995) | √ | √ |  | √ |  |  |
| Stryckman (1996) | √ |  |  |  |  | - responsible and empowered local communities |
| Criel 1998 | √ | √ | √ | √ | √ | - Impact on demand - Impact on utilization - Impact on supply - Impact on quality of care - Impact on community participation - accountability |
| Murray & Frenk (1999) | √ | √ |  | √ |  | - Effect on citizen participation - Effect on multisectoral action |
| V. Richard 2004 |  | √ | √ |  |  | - Overall level of government spending - cost recovery - care network - Private contributions - payment mechanisms - Equity of care (28) |
| Didier Gobbers 2004 | √ |  | √ |  |  | - Transparency and accountability (29) |
| IFC 2007 | √ | √ | √ | √ |  | - Overall level of government spending - Private sector contribution - Importance of private sector contributions |
| McIntyre 2007 | √ | √ | √ | √ |  |  |
| Kutzin et al. 2010 | √ | √ |  | √ |  | - Financial risk protection - Quality - Transparency and accountability |
| United Nations Foundation and  The Global Fund 2014 |  |  | √ | √ |  | - macro-economic feasibility and stability |
| CABRI 2016 | √ |  | √ |  |  | - Guidance Administration - Efficiency (30) |
| Garand et al 2016 | √ | √ | √ |  |  | - Financial risk protection |
| McIntyre & Kutzin (2016) | √ | √ |  | √ |  | - Financial risk protection - Extent of fragmentation |
| GIZ 2019 | √ |  |  | √ |  | - Sanitary situation - Performance of elements of the health system |
| Doherty et al. 2019 | √ | √ | √ | √ |  | - Financial risk protection - Risk cross-subsidization |
| Jowett et al. (2020) |  | √ | √ |  |  | - incentives for healthier behavior by individuals and firms - Health expenditure is based predominantly on public/compulsory funding sources |
